# The International Hahnemannian Association—Address to Members

**Published:** 1895-02

**Authors:** A. R. Morgan

**Affiliations:** Chairman of Board of Censors; Waterbury, Ct.


### THE INTERNATIONAL HAHNEMANNIAN ASSOCIATION—ADDRESS TO MEMBERS.

Owing largely to the unhomœopathic teachings of many of our "Homœopathic Medical Colleges," the ratio of increase of Hahnemannian practitioners has not kept pace with the rapid growth in numbers of the so-called homœopathic physicians; but in spite of this serious adverse influence, there are evidences on every hand, both among physicians and laymen, of a demand for purer doctrines and a more loyal practice.

The establishment of flourishing *Organon* Societies in many places are among the cheering indications.

Are we, as members of this Association, from whom much is expected, doing all we can to meet this demand?

Does our duty to the cause end with fidelity to our declaration of principles? Is it not also incumbent upon us to take the lead, to do our best toward bringing together in the I. H. A., so far as possible, every sound Hahnemannian, many of whom have not yet been enrolled as members, but who should stand shoulder to shoulder with us in this crusade of "the faithful" against eclecticism, masquerading under the guise of Homœopathy?

Let us make no delay in obtaining applications for *Active Membership*, remembering that such applications must be in the hands of the Chairman of the Board of Censors at least six months prior to the next annual meeting.

Now for missionary work! Many of those who to-day are involved in the uncertainties of a mixed practice, have been led astray by false teachings; they have had no fair opportunity to know even the logical principles of our art of cure, nor to witness the superior results of a genuine homœopathic practice.

Many of them, sincere and honest men, dissatisfied with their present experiences, are longing for a better way, and our Association would be to them an *educational* institution. It needs but your personal solicitation to induce many of them to apply for *Junior Membership;* where, without committing themselves in advance to our declaration of principles, they would be able to secure the benefits to be derived from attending our meetings, listening to our papers, participating in our discussions, and, in fact, entitled to nearly all the rights and privileges of full membership. Ultimately, many of these Juniors will be saved to the cause, and become Active members.

Applications for Junior Membership may be obtained now, by applying for the same to

A. R. MORGAN,
*Chairman of Board of Censors.*

WATERBURY, CT., November 24th, 1894.

